# A gland-sparing, intraoral sialolithotomy approach for hilar and intraparenchymal multiple stones in the submandibular gland

**DOI:** 10.1038/s41598-020-65519-7

**Published:** 2020-05-22

**Authors:** Huan Shi, Jun Zhao, Eugene Poh Hze-Khoong, Shixin Liu, Xuelai Yin, Yongjie Hu

**Affiliations:** 1 Department of Oral and Maxillofacial-Head & Neck Oncology, Shanghai Ninth People’s Hospital, College of Stomatology, Shanghai Jiao Tong University School of Medicine, National Clinical Research Center for Oral Disease; Shanghai Key Laboratory of Stomatology & Shanghai Research Institute of Stomatology, Shanghai, China; 2 Department of Oral Surgery, Shanghai Ninth People’s Hospital, College of Stomatology, Shanghai Jiao Tong University School of Medicine; National Clinical Research Center for Oral Disease, Shanghai Key Laboratory of Stomatology & Shanghai Research Institute of Stomatology, Shanghai, China; 3 Department of Prosthodontics, Shanghai Ninth People’s Hospital, College of Stomatology, Shanghai Jiao Tong University School of Medicine; National Clinical Research Center for Oral Disease, Shanghai Key Laboratory of Stomatology & Shanghai Research Institute of Stomatology, Shanghai, China; 4 Department of Oral and Maxillofacial Surgery, Khoo Teck Puat Hospital, Yishun, Singapore; 5 Department of Oral and Maxillofacial Surgery, Zhengzhou Second People’s Hospital, Zhengzhou, China

**Keywords:** Oral diseases, Salivary gland diseases

## Abstract

Multiple intraglandular sialolithiasis for stones deep in the glandular parenchyma may require submandibulectomies, especially if sialendoscopic facilities are unavailable. We describe a gland-sparing intraoral sialolithotomy approach for both hilar and intraparenchymal multiple sialoliths. Nine patients with obstructive sialadenitis resulting from multiple sialoliths in both the deep hilar region and the submandibular gland parenchyma were selected for this study. Ultrasonography and computer tomography (CT) scans were performed to determine the location, number and sizes of the calculi and the distance between hilar and intraparenchymal sialoliths. All sialoliths were removed via gland-sparing, intraoral sialolithotomy. In all, 27 stones were found in the 9 patients. The hilar and deeper sialoliths were 4.5–11 and 0.8–4.5 mm, respectively, in diameter. The largest distance between the hilar and intraparenchymal sialoliths was 28.3 mm. Sialoliths in the hilar region were excised through an intraoral incision before deeper intraparenchymal stones were eased out of the same incision site. Postoperative follow-up imaging verified complete sialolith removal. Therefore, submandibular gland multiple sialoliths in the hilum and parenchyma can be successfully removed via an intraoral sialolithotomy under general anesthesia, thereby preserving the gland and restoring its secretory function.

## Introduction

Sialolithiasis is the most common cause of obstructive sialadenitis and occurs in approximately 1.2–1.5% of the overall population^1^. In 80–90% of reported cases, these calculi are found within the submandibular gland^2,3^. Koch *et al*. reported that 34% of sialoliths are distributed in the distal duct (ductal stones), 57% within the hilum of the gland (hilar stones), and 9% in the gland parenchyma (intraparenchymal stones)^4^. In these previous studies, the authors propose that the turbulent salivary flow in these 3 regions leads to the formation of mucous plugs that act as niduses for the deposition of inorganic and organic substances, resulting in the formation of sialoliths.

Treatment requires the removal of the sialoliths, and various methods to do so have been described in the literature. Stones in the distal duct are often palpable and can be easily retrieved via an intraoral sialolithotomy under local anesthesia. The advent of sialendoscopy-assisted intraoral sialolithotomy reduced the prevalence of submandibulectomies as the default treatment modality for deeper, nonpalpable stones in the duct and hilum^5,6^. It has been reported that over 95% of deep hilar stones in the submandibular gland can be endoscopically removed^4,7^, while for larger stones, laser-beam fragmentation and extracorporeal shock-wave lithotripsy (ESWL) had a 75% effective rate and allowed for the complete retrieval of stones in half of the cases^6,8–10^. However, deeper intraparenchymal stones that are inaccessible to sialendoscopy remain a problem, and previous authors have reported that 5–10% of these patients cannot be conservatively treated and should therefore be considered for submandibulectomy as the definitive treatment of choice^5,6,10^. Furthermore, sialendoscopy and ESWL may not always be readily available due to the need for subspecialization training and the prohibitive costs of procurement and equipment maintenance. Both treatment modalities could also cause inadvertent fragmentation and retrograde displacement of ductal and hilar sialoliths deeper into the gland parenchyma.

We postulate that intraoral excision of palpable hilar stones could permit the passage of smaller intraparenchymal stones through the same incision site in tandem with extraoral gland massage when performed under general anesthesia, thus avoiding the need for submandibulectomies that are otherwise indicated in such instances.

## Material and methods

### Patients

In all, 104 patients with various submandibular gland sialoliths were treated at the Shanghai Ninth People’s Hospital, China, between October 2018 and May 2019. Of these, only 9 met the inclusion criteria and consented to the study, which was approved by the Shanghai Jiao Tong University School of Medicine Affiliated Ninth People’s Hospital’s Ethics Committee and Review Board.

The study’s inclusion criteria included patients with 1) ultrasonographic and/or computed tomographic verification of multiple submandibular sialoliths, 2) two or more sialoliths located in both the hilar and intraparenchymal regions, 3) at least one palpable stone in the hilum or deep hilar region, 4) the intraparenchymal sialoliths with a maximum diameter of 5 mm, and 5) no history of previous surgical management of the sialoliths.

Salivary flow rates were assessed in each patient using the Saxon Test. Patients chewed on a folded sterile sponge for 2 minutes, and the saliva-soaked sponge was then weighed and compared against its original weight. Computed tomography was also performed in each patient to quantify and determine the exact location of the sialoliths and measure their respective sizes and relative distances from one another.

### Surgical technique

All 9 patients were operated on under general anesthesia by the same surgical team. Anesthetic drugs were standardized for all 9 patients as follows: penehyclidine hydrochloride, 0.01 mg/kg; midazolam, 0.05 mg/kg; fentanyl, 2 µg/kg; propofol, 1–2 mg/kg; cisatracurium, 0.2 mg/kg; and 1.5–2.5% sevoflurane and 0.01–0.1 µg/kg/min remifentanil infusion for maintenance.

**Figure 1:**
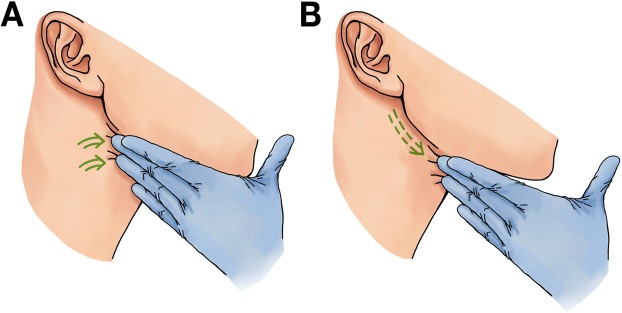
Continuous extraoral massage was applied to the submandibular gland in a posterior-anterior direction.

The palpable stones in the deep hilar regions were first accessed via a mucosal incision made immediately overlying the associated Wharton’s duct in the floor of the mouth. Blunt dissection was performed to retract a mucosal flap and expose the duct while taking care to identify and preserve the lingual nerve crossing the duct latero-medially at the posterior aspect. The application of firm, upwards digital pressure at the submandibular triangle medial to the inferior border of the mandible resulted in the bulging of the medial portion of the gland from the floor of the mouth, thereby enabling the palpation of the calculi within. A direct linear incision was made over the roof of the duct to expose the hilar stone for easy retrieval. Continuous extraoral massaging of the submandibular gland in a posterior-anterior circuitous motion elicited large amounts of salivary drainage from the incision site, leading to the expulsion of the intraparenchymal sialoliths (Fig. 1). All stones were accounted for before each duct was copiously flushed with normal saline. The incised ducts were either left unrepaired beneath a loosely closed mucosa or kept open by sewing their incised edges to the overlying mucosal incision for the purpose of creating a new orifice for salivary drainage.

### Ethical approval

All procedures performed in studies involving human participants were conducted in accordance with the ethical standards of Shanghai Ninth People’s Hospital’s Ethics Committee and Review Board and with the 1964 Helsinki Declaration and its later amendments or comparable ethical standards.

### Informed consent

Informed consent was obtained from all individual participants included in the study.

## Results

The 9 patients included 7 males and 2 females aged 22–43 (mean, 32.2 years). Five of these patients were smokers (n = 5 [55.6%]), and the reported duration of sialadenitis ranged from 2–18 months (mean 12.5 months). Common clinical presentations included pain (n = 9 [100%]), swelling (n = 7 [77.8%]), preprandial symptoms (n = 4 [44.4%]) and xerostomia (n = 1 [11.1%]). None reported taste impairment or oral dysesthesia (n = 0 [0%]) **(**Table 1**)**. The results indicated that xerostomia and taste impairment are unspecific and atypical symptoms of obstructive salivary gland diseases.

**Table 1:** Patient Demographics and Symptoms.

Variable	Value
Age, mean (range), year	32.2 (22–43)
**Gender**	
Male	7 (77.8%)
Female	2 (22.2%)
Symptom duration, mean (range), month	12.5 (2–18)
Pain	9 (100%)
Swelling	7 (77.8%)
Preprandial symptoms	4 (44.4%)
Xerostomia	1 (11.1%)
Antibiotics	8 (88.9%)
Smoking	5 (40%)
Partiality to a particular kind of food	0 (0%)

Values are presented as numbers (%) unless otherwise indicated.

All 9 patients had normal saliva production in the initial Saxon test (mean, 3.76 g/2 min; range, 2.7–5.2 g/2 min). The average size of the hilar sialoliths was 7.4 ± 3 mm (range 4.5–11 mm), while the intraparenchymal sialoliths averaged 3.2 ± 1.1 mm (range 0.8–4.5 mm). The average distance between the hilar and intraparenchymal sialoliths was 15.6 ± 12.5 mm (range 3.9–28.3 mm). The average length of hilar sialoliths was 7.4 ± 3 (range, 4.5–11) mm and that of the parenchyma sialolith was 1.5 ± 1.1 (range, 0.8–2.2) mm. The locations of the sialoliths are shown in Table 2.

**Table 2:** Summary of the measured variables.

Variable	Value
Saxon test, mean (range), g/2 min	3.76 (2.7–5.2)
Average size of hilar sialoliths, mean (range), mm	7.4 (4.5–11)
Average size of intraparenchymal sialoliths, mean (range), mm	3.2 (0.8–4.5)
Average distance between the hilar and intraparenchymal sialoliths, mean (range), mm	15.6 (3.85–28.28)
**Location of sialoliths, Number (%)**	
Left side	6 (66.7%)
Right side	3 (33.3%)
Hilum stone	9 (100%)
**Intraglandular stone**	
One stones	2 (22.2%)
Two stones	5 (55.6%)
Three stones	2 (22.2%)

The average surgical time was 32 ± 10.8 minutes (range 24–43 minutes), and all patients had an uneventful postoperative hospitalization of no more than 2 days before being discharged with a 2-week course of Pilocarpine (10 mg thrice daily) and Vitamin C lozenges. All patients were closely followed up at 2 weeks, 3 months and 12 months. Four patients complained of pain and 2 complained of mild swelling 2 weeks after surgery. A small amount of purulent exudate was observed in one patient at 2 weeks postoperative. All complications were alleviated by rational drug use or extraoral massage of the submandibular gland at 3 months postoperative. There were no complaints of lingual paresthesia. All 9 patients reported complete resolution of their initial complaints and presenting symptoms at 3 months postoperative. Submandibular gland ultrasonography was performed for each patient at the 3rd month review to verify the absence of any remaining sialoliths. Due to the COVID-19 pandemic, all 9 patients were reached via phone call for a 12-month follow-up assessment. Only one patient complained of mild pain twice after surgery intervention. **(**Table 3**)**.

**Table 3:** Follow-up data.

Characteristic	2 weeks, No. (%)	3 months, No. (%)	1 year, No. (%)
Lingual paresthesia	0 (0%)	0 (0%)	0 (0%)
**Pain**			
Severe	0 (0%)	0 (0%)	0 (0%)
Moderate	2 (22.2%)	0 (0%)	0 (0%)
Mild	2 (22.2%)	0 (0%)	1 (11.1%)
**Swelling**			
Severe	0 (0%)	0 (0%)	0 (0%)
Moderate	0 (0%)	0 (0%)	0 (0%)
Mild	2 (22.2%)	0 (0%)	0 (0%)
Xerostomia	0 (0%)	0 (0%)	0 (0%)
Purulent exudate	1 (11.1%)	0 (0%)	NA
Remaining sialolith	NA	0 (0%)	NA

### Case presentation

#### Case 1

**Figure 2:**
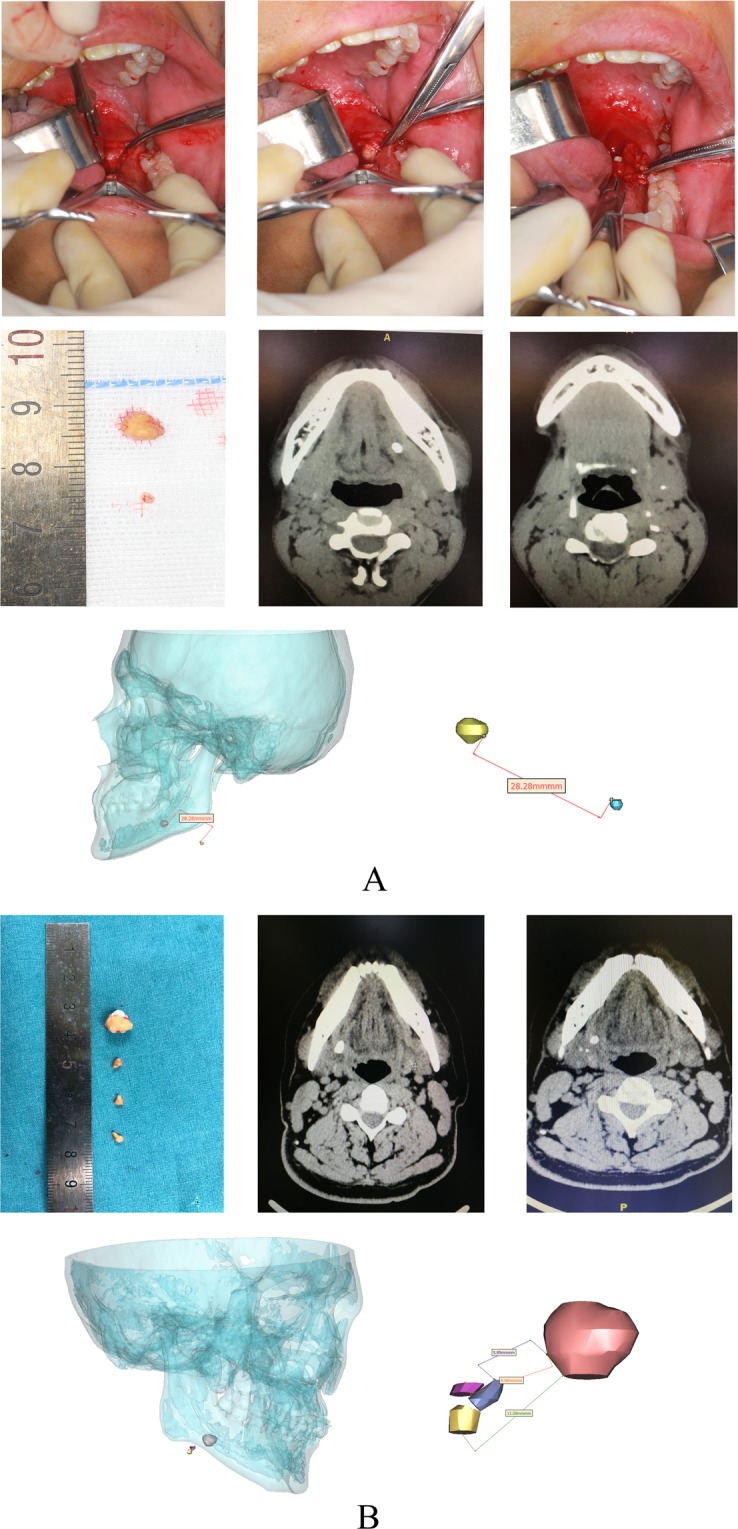
Intraoperative photographs and CT three-dimensional reconstructions of Case 1 and Case 2.

A 23-year-old man presented with recurrent and painful left-sided submandibular swelling for over a month. A CT scan revealed the presence of one large hilar stone and a separate smaller stone deep in the parenchyma of the gland. The CT 3-dimensional reconstructed image showed that there was a distance of 28.28 mm between the hilar and intraparenchymal sialoliths. Both stones were successfully retrieved via a transoral sialolithotomy in which the larger stone was directly excised before the smaller stone was eased out of the same incision site via submandibular massage. **(**Fig. 2A**)**.

#### Case 2

A 47-year-old man presented recurrent right-sided painful swelling in the submandibular region on 18 May 2018. Multiple stones in the glandular parenchyma were visualized on CT scans, in which 1 large stone was observed to be lodged in the hilum and 3 smaller stones within the parenchyma. A CT 3-dimensional reconstructed image showed that the distances between the hilar stone and the 3 intraparenchymal sialoliths were 11.09, 8.58 and 5.95 mm. A transoral sialolithotomy was performed to remove the hilar stone before the 3 smaller parenchymal stones were expunged from the same incision site after extraoral massage of the gland. **(**Fig. 2B**)**.

## Discussion

The prevalence of sialoliths in the submandibular gland can be attributed to multiple factors, such as the gland’s complex anatomical structure and ductal system; the presence of an intervening mylohyoid muscle that separates the larger lateral portion from the smaller medial segment; anti-gravitational salivary flow through the narrow, long and winding Wharton’s duct; and the mucoid salivary component from this mixed sero-mucous gland. Marchal *et al*. suggested that the presence of a sphincter in the first 3 cm of the duct could result in the retrograde migration of various organic and inorganic ingredients, a process that could contribute to the formation of sialoliths^11^.

Classical symptoms of submandibular sialolithiasis include swelling and pain over the gland that is exacerbated during meals as well as gritty saliva, a painful lump in the floor of the mouth, and minimal salivary discharge from the associated duct of the orifice. Mandibular occlusal films and dental panoramic radiographs are often the first forms of diagnostic imaging used for visualization of calcified deposits, while computer tomography (CT) and magnetic resonance imaging (MRI) offer greater definition and the ability to exclude other differential diagnoses. High-resolution ultrasonography is a comparatively simpler and less expensive alternative with a reportedly higher sensitivity than can be achieved using CT and MRI^12,13^. MRI has been demonstrated to be unsuitable for sufficiently diagnosing smaller calculi. Definitive management usually commences after the acute inflammatory phase has been suppressed by a course of anti-inflammatory medications and broad-spectrum antibiotics, and various treatment modalities are available, depending on the location, number and sizes of the sialoliths.

Stones in the anterior duct are usually palpable and can be easily excised under local anesthesia via a direct cut-down procedure. Palpable stones in the hilum can be similarly approached, but this should preferably be done under sedation due to the significant discomfort involved. Intraparenchymal stones are often too deeply located and are consequentially unamenable to direct intraoral access. Moreover, they are usually much smaller in size and may be conservatively managed with saliva-inducing agents, such as parasympathomimetics (e.g., pilocarpine) or anti-cholinesterases (e.g., neostigmine), to encourage their distal migration into Wharton’s duct. Minimally invasive procedures, such as intervention sialendoscopy, ESWL and combined endoscopic-surgical procedures, are gaining popularity as the first-line treatment option in these patients^6,14^ as they significantly reduce the need for submandibulectomies^15^. However, these endoscopic procedures are technically challenging and have variable success and complication rates, and centers lacking appropriate facilities will invariably have to resort to conventional submandibular gland resection. The dilemma of whether to spare or resect the entire gland is further compounded by the simultaneous occurrence of other sialoliths that are palpable intraorally and hence amenable to a conservative, gland-preserving approach.

Saliva production and composition are regulated by the autonomic nervous system, via which sympathetic innervation results in an overall decrease in both saliva production and blood flow to the glands and an increase in protein secretion. The parasympathetic system plays a more important role in saliva production as the release of acetylcholine onto muscarinic M3 receptors results in increased production of watery saliva by acinar cells. The combined effects of increased blood flow and contraction of the myoepithelium from parasympathetic innervation further amplify the saliva expulsion rate. In patients under general anesthesia, the suppression of sympathetic activity by inhalational anesthetic agents intraoperatively tilts the balance towards a parasympathetic predominance, leading to an increase in saliva production^16^. We propose that removing the hilar stones at the onset releases the built-up pressure within the obstructed gland, permitting the drainage of saliva along with the smaller intraparenchymal sialoliths. Furthermore, the use of muscle relaxants in general anesthesia may also relax the mylohyoid muscle that the submandibular gland is wrapped over, potentially permitting easier passage of the smaller intraglandular sialoliths towards the hilum. The application of circuitous, posterior-anterior extraoral submandibular gland massage serves to encourage anti-gravitational salivary flow from the inferiorly located gland into the medial-superiorly located Wharton’s duct.

The largest intraparenchymal stone in our study measured 4.5 mm in diameter, and it remains to be seen whether larger stones would be amenable to this approach. We recommend that ESWL be considered instead for the fragmentation of larger intraparenchymal sialoliths followed by the use of saliva-inducing medications to encourage their spontaneous discharge. Alternatively, a combination of ESWL and intraoral sialolithotomy may be considered before proceeding with a conventional submandibulectomy. Further studies comprising larger sample sizes as well as quality of life surveys comparing patients who underwent these procedures versus those who underwent submandibulectomies would be useful to validate the viability of this treatment option.

## Conclusion

Approximately 90% of all submandibular gland sialoliths are located within the Wharton duct, up to the hilum of the gland. Deeply located intraparenchymal stones are generally smaller and account for the remaining 10%. Management options for these sialoliths include medications, lithotripsies, sialendoscopies or submandibulectomies^7^. In light of the variable success of these approaches, intraoral sialolithotomy is a viable treatment modality for patients with simultaneously occurring deep hilar and intraparenchymal sialoliths.
